# Momordin Ic, a new natural SENP1 inhibitor, inhibits prostate cancer cell proliferation

**DOI:** 10.18632/oncotarget.10636

**Published:** 2016-07-16

**Authors:** Jingjing Wu, Hu Lei, Jinfu Zhang, Xiangyun Chen, Caixia Tang, Weiwei Wang, Hanzhang Xu, Weilie Xiao, Wenli Gu, Yingli Wu

**Affiliations:** ^1^ Department of Clinical Laboratory, Shanghai Ninth People's Hospital, Shanghai Jiao Tong University School of Medicine, Shanghai, China; ^2^ Hongqiao International Institute of Medicine, Shanghai Tongren Hospital/Faculty of Basic Medicine, Chemical Biology Division of Shanghai Universities E-Institutes, Key Laboratory of Cell Differentiation and Apoptosis of The Chinese Ministry of Education, Shanghai Jiao Tong University School of Medicine, Shanghai, China; ^3^ State Key Laboratory of Phytochemistry and Plant Resources in West China, Kunming Institute of Botany, Chinese Academy of Sciences, Yunnan, China

**Keywords:** Momordin Ic, SENP1, SUMOylation, proliferation, prostate cancer

## Abstract

SUMO-specific protease 1 (SENP1), a member of the de-SUMOylation protease family, is elevated in prostate cancer (PCa) cells and is involved in PCa pathogenesis. Momordin Ιc (Mc), a natural pentacyclic triterpenoid, inhibited SENP1 *in vitro*, as reflected by reduced SENP1C-induced cleavage of SUMO2-ΔRanGAP1. Mc also altered the thermal stability of SENP1 in a newly developed cellular thermal shift assay, indicating that Mc directly interacts with SENP1 in PCa cells. Consistent with SENP1 inhibition, Mc increased SUMOylated protein levels, which was further confirmed by the accumulation of two known SUMOylated proteins, hypoxia inducible factor-1a and nucleus accumbens associated protein 1 in PC3 cells. Compared to LNCaP and normal prostate epithelial RWPE-1 cells, PC3 cells had higher levels of SENP1 mRNA and were more sensitive to Mc-induced growth inhibition. Mc also reduced SENP1 mRNA levels in PCa cells. Overexpression of SENP1 rescued PC3 cells from Mc-induced apoptosis. Finally, Mc suppressed cell proliferation and induced cell death *in vivo* in a xenograft PC3 tumor mouse model. These findings demonstrate that Mc is a novel SENP1 inhibitor with potential therapeutic value for PCa. Investigation of other pentacyclic triterpenoids may aid in the development of novel SENP1 inhibitor drugs.

## INTRODUCTION

Prostate cancer (PCa) is the most frequently diagnosed cancer, and the second leading cause of cancer-related death, in men, with an estimated 27,540 deaths worldwide in 2015 [1]. Recently, the use of the prostate-specific antigen (PSA) blood test for screening has dramatically increased PCa incidence rates in China [2]. Despite advancements in radiation therapy, chemotherapy, surgery, hormone (androgen deprivation) therapy, and combined treatments, patient outcomes vary depending on cancer stage and grade as well as patient characteristics such as age, other medical conditions, and treatment preferences. Additionally, patients who develop fatal hormone-refractory disease are limited to docetaxel-based chemotherapy, which extends survival just by a few months [3]. Therefore, it is crucial to develop novel strategies to treat prostate cancer.

SUMOs are highly-conserved 11 kDa proteins that covalently attach to proteins to modulate their functions. Mammalian cells express three major SUMO paralogues: SUMO1, SUMO2 and SUMO3. SUMOylation regulates many cellular processes, including protein-protein interactions, transcription, protein localization, cell cycle progression, DNA replication and repair, chromatin organization, and RNA metabolism [4–6]. SUMOylation is catalyzed by SUMO-specific activating (E1), conjugating (E2), and ligating (E3) enzymes [7]. SUMO-specific proteases (SENPs), which include 6 members named SENP1, SENP2, SENP3, SENP5, SENP6, and SENP7, reverse SUMOylation. SENP1 is a nuclear protease that, when overexpressed, deconjugates a large number of SUMOylated proteins. Altered expression of SENPs is observed in several carcinomas [8–10]. Specifically, SENP1 plays an important role in the occurrence [11], development [12, 13], and metastasis [14] of PCa. Therefore, SENP1 is considered a potential therapeutic target for PCa.

Natural products play an important role in pharmacological research [15]. It is estimated that about 40% of all medicines are natural products or semi-synthetic derivatives of natural products. Despite competition from other drug discovery methods, natural products are still an important source of new drugs. However, none of the currently identified SENP1 inhibitors [16–18] is derived from natural compounds, nor have these inhibitors been tested in preclinical models. One goal of this study is to identify natural products with SENP1 inhibitory activity.

Momordin Ιc (Mc) is a pentacyclic triterpene that has been extracted from various Chinese and Japanwese natural medicines, such as the dried fruit of *Kochia scoparia* (L.) [19]. Mc accelerates gastrointestinal transit (GIT) [20, 21], inhibits gastric emptying [22], inhibits ethanol-induced gastric mucosal lesions [23], and reduces carbon tetrachloride-induced hepatotoxicity in rats [24]. Mc also has anticancer activity against HepG2 [25, 26]. However, the underlying mechanisms of these effects are largely unknown.

In this study, by screening a small library of natural compounds, we identified Mc as novel SENP1 inhibitor that inhibited proliferation of prostate cancer cells *in vitro* and *in vivo*. Mc represents a promising candidate for the development of SENP1 inhibitors with pentacyclic tripernoid scaffolds.

## RESULTS

### Mc inhibits SENP1 activity *in vitro*

**Figure 1:**
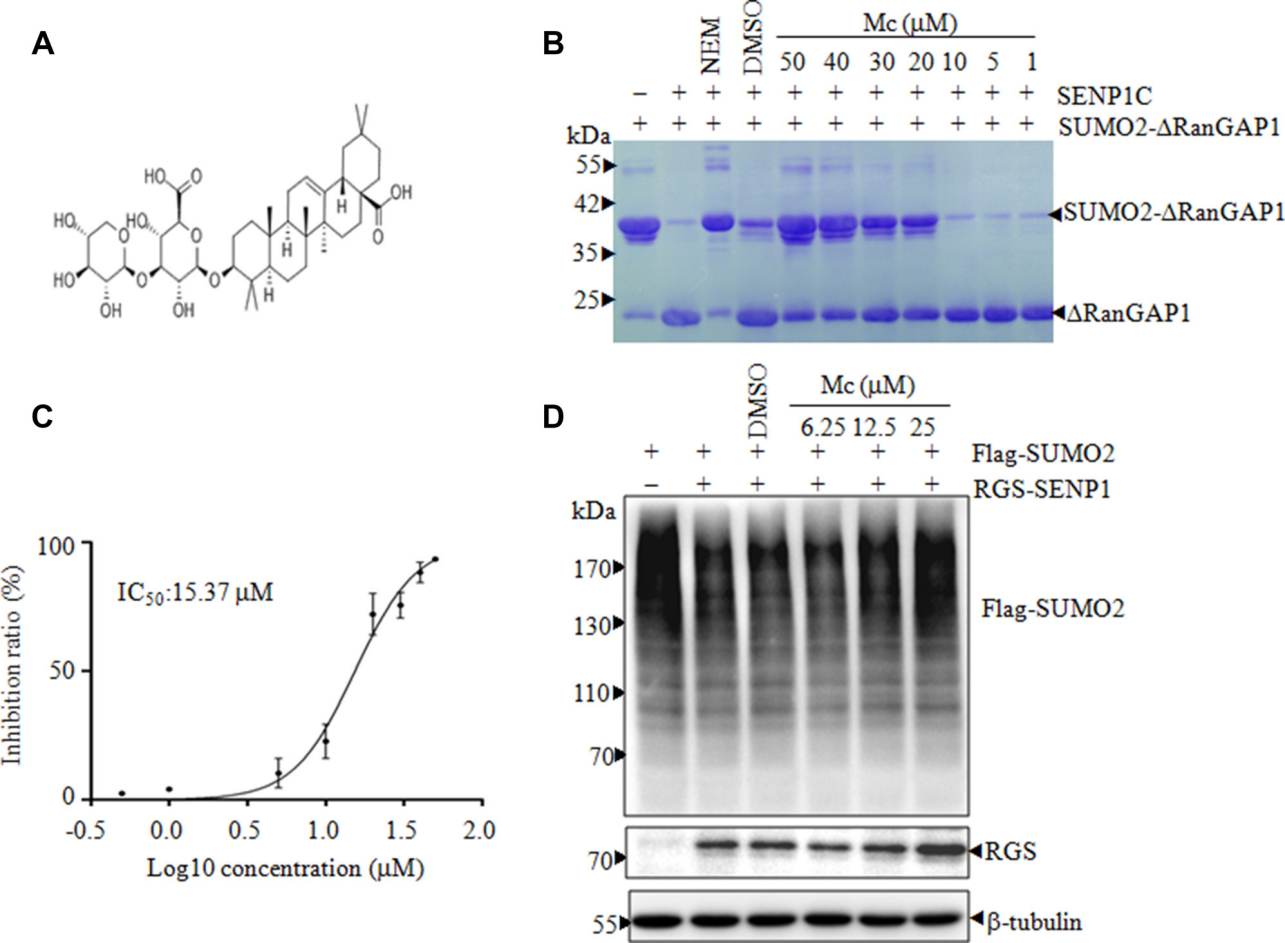
Mc is a SENP1 inhibitor (**A**). The chemical structure of Mc. (**B**). In an *in vitro* gel-based SENP1 activity assay, various concentrations of Mc were preincubated with 20 nM SENP1C before SUMO2-ΔRanGAP1 was added. After incubation, the reactions were stopped and the products were separated by 12% SDS-PAGE and visualized with coomassie brilliant blue (G250). NEM stands for N-Ethylmaleimide, an irreversible inhibitor of all cysteine peptidases. (**C**). After the *in vitro* gel-based SENP activity assay, gray scanning analysis was carried out using ImageJ software, and a curve was fitted using GraphPad Prism 5.0 after three independent experiments. The IC_50_ of Mc was 15.37 μM. (**D**). HEK293T cells were transiently transfected with Flag-SUMO2 and empty vector or RGS-SENP1 for 24 h and then treated with DMSO or 6.25, 12.5, or 25 μM Mc for 2 h; the indicated proteins were detected by Western blotting.

In a screening to identify natural compounds with SENP1 inhibitor activity, we found that Mc (Figure 1A), a natural pentacyclic triterpenic compound, markedly inhibited SENP1C-mediated cleavage of SUMO2-ΔRanGAP1 in an *in vitro* deSUMOylation assay (Figure 1B). The IC_50_ of Mc-induced SENP1C inhibition was 15.37 μM (Figure 1C). As only SENP1C contained the appropriate catalytic domain, we next examined whether Mc inhibited the activity of full-length SENP1 in cells. To this end, HEK293T cells were transfected with full-length SENP1 and Flag-tagged SUMO2 and then treated with Mc. As shown in Figure 1D, the accumulation of SUMO-modified proteins increased as the Mc treatment concentration increased, indicating that Mc inhibits the isopeptidase activity of full-length SENP1 in cells.

### Mc interacts with SENP1 in cells

**Figure 2:**
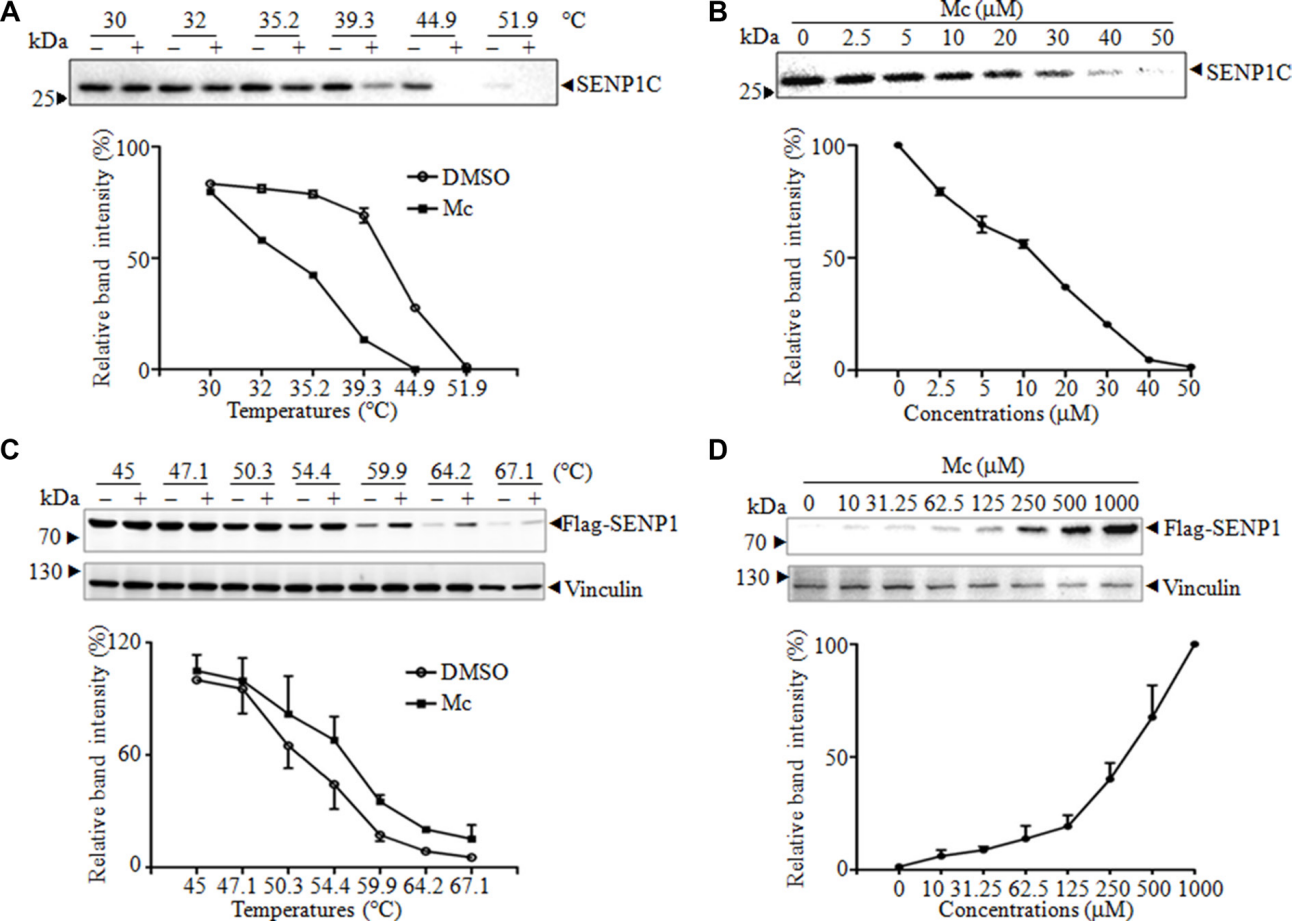
Mc alters SENP1 thermal stabilization (**A**–**B**). Four μg of purified SENP1C was incubated with 50 μM Mc at the indicated temperatures (A), and 4 μg purified SENP1C was incubated with indicated concentrations of Mc at 45°C for 3 min (B). After centrifugation, supernatant was analyzed by western blot with anti-SENP1 antibody and bands were scanned for densitometric analysis. The thermal melt curve (A) and the isothermal dose-response fingerprint (B) are shown. (**C**–**D**). Lysate from PC3 cells stably transfected with pBabe-Flag-SENP1 was treated with 100 μM Mc at the indicated temperatures (C) or with the indicated concentrations of Mc at 60°C for 3 min (D), then analyzed by western blot with anti-flag antibody. The bands were scanned for densitometric analysis, and the thermal melt curve (C) and the isothermal dose-response fingerprint (D) are shown.

Because Mc inhibited the activity of SENP1 *in vitro*, we next investigated whether Mc directly binds to SENP1 using the cellular thermal shift assay (CETSA). CETSA, a newly-developed method for evaluating drug binding to target proteins in cells and tissue samples, is based on the biophysical principle of ligand-induced thermal stabilization of target proteins [27]. First, we performed a thermal shift assay with purified SENP1C. Compared to DMSO, Mc markedly decreased the thermal stability of SENP1C at the temperatures examined (Figure 2A). Moreover, Mc decreased the accumulation of SENP1C in a dose-dependent manner (Figure 2B). These results indicate that Mc directly altered the thermal stability of SENP1C. Consistent with this result, the drug affinity responsive target stability (DARTS) assay showed that Mc protected SENP1C from pronase-induced proteolysis, indicating a direct interaction between Mc and SENP1C *in vitro* (Supplementary Figure S1). Next, we used CETSA to evaluate the interaction of SENP1 with Mc in androgen receptor-negative prostate cancer PC3 cells. As the commercially available SENP1 antibody did not reliably detect endogenous SENP1, we transfected Flag-tagged SENP1 into PC3 cells (PC3^Flag-SENP1^). As shown in Figure 2C, compared to DMSO, Mc markedly increased the accumulation of Flag-SENP1 in the soluble fraction at the temperatures examined. We also tested whether Flag-SENP1 stability during heating depended on the dose of Mc. As shown in Figure 2D, Flag-SENP1 accumulation markedly increased as Mc concentration increased. As a negative control, we demonstrated that Mc did not increase the stability of vinculin in cells. These data suggest that Mc directly interacts with SENP1 in cells.

### Mc increases SUMOylated protein levels in prostate cancer cells

**Figure 3:**
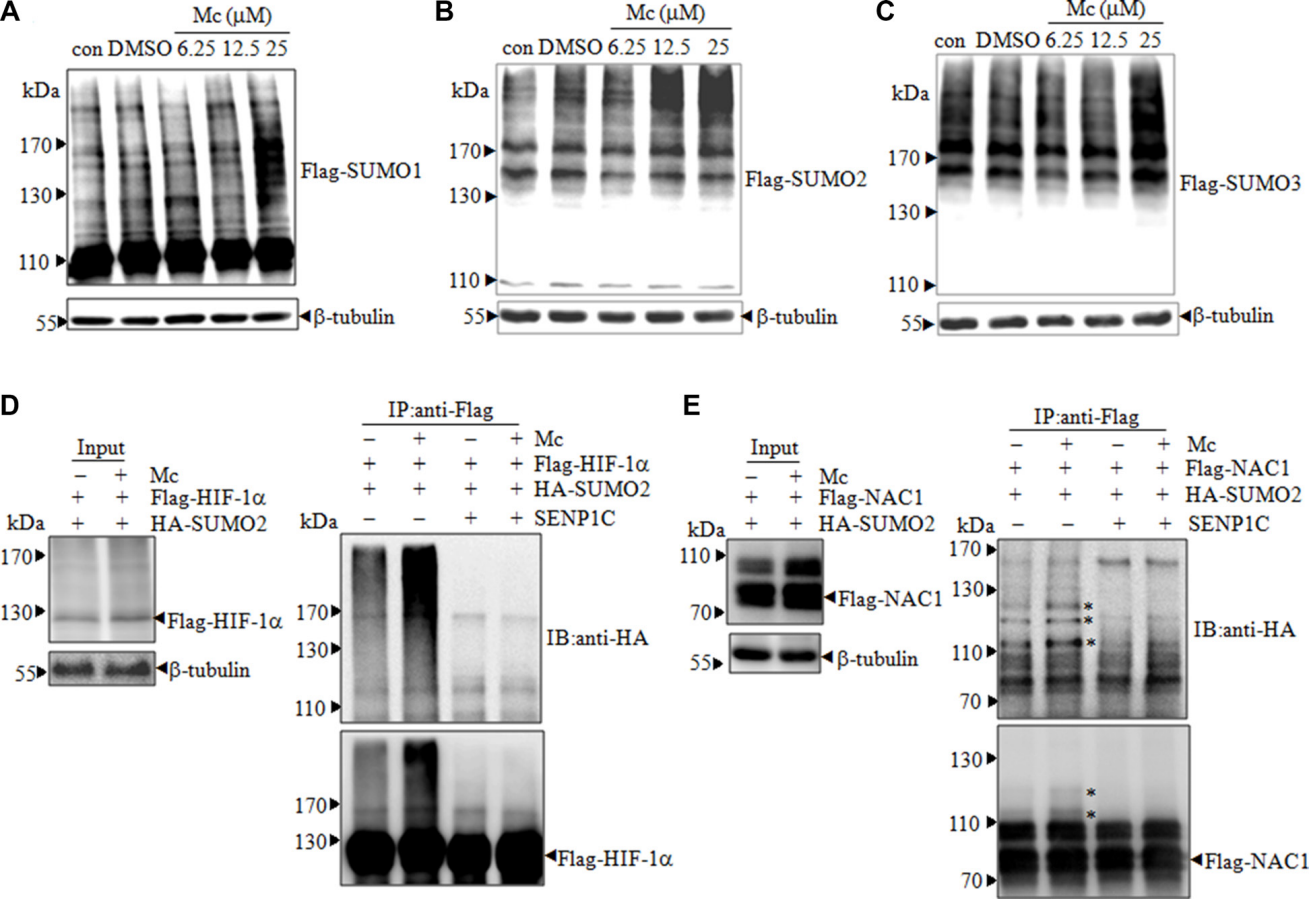
Mc induces accumulation of SUMO-conjugated proteins in PC3 cells (**A**–**C**). PC3 cells stably expressing Flag-tagged SUMO1 (A), SUMO2 (B), and SUMO3 (C) were treated with DMSO or 6.25, 12.5, or 25 μM Mc for 1 h (A) or 2 h (B and C). The indicated proteins were detected by western blotting with anti-flag antibody. (**D**–**E**). PC3 cells were transfected with plasmids encoding HA-SUMO2 and Flag-HIF-1α (D) or NAC1 (E). After 48 h, cells were treated with 20 μM Mc for 2 h (cells transfected with HIF-1α were pretreated with 5 μM MG132 for 4 h). Flag-HIF-1α or Flag-NAC1 was immunoprecipitated from cell lysates and then subjected to western blot in the presence or absence of purified SENP1C. * indicates SUMOylated NAC1.

Given that Mc inhibits SENP1 activity *in vitro* and interacts with SENP1 in cells, Mc likely inhibits SENP1 activity in PC3 cells. Because the intracellular concentration of SUMO1 is low and less dynamic in PC3 cells, and because there are no specific antibodies to distinguish endogenous SUMO2 from SUMO3, we stably transfected PC3 cells with pBabe-Flag-SUMO1/2/3 plasmids (PC3^Flag-SUMO1/2/3^) to increase the pool of free SUMO1 and to distinguish between proteins modified with SUMO2 or SUMO3. 25 μM Mc treatment induced a large increase in SUMOylated protein levels in SUMO2-transfected PC3 cells (PC3^Flag-SUMO2^) (Figure 3B) and a moderate increase in SUMO1/3-transfected PC3 cells (PC3^Flag-SUMO1/3^) (Figure 3A and 3C), as indicated by the appearance of smeared high molecular weight bands. These results suggest that Mc inhibits the isopeptidase activity of endogenous SENP1 and subsequently leads to the accumulation of SUMOylated proteins. To further confirm that Mc inhibits SENP1 activity, we examined whether Mc altered the SUMOylation of the known SUMO substrates HIF-1α and nucleus accumbens-associated protein 1 (NAC1). HIF-1α is a well-known and important oncogene in PCa [28]. NAC1 is associated with pathogenesis in several types of cancer cells [29–31], and we previously identified NAC1 as a possible SUMO substrate in PCa cells [16]. PC3 cells were transiently transfected with Flag-HIF-1α and HA-SUMO2 and then treated with Mc for 2 hours. Flag-HIF-1α was immunoprecipitated from cell lysate and SUMOylation status was detected by western blot. As shown in Figure 3D, Mc treatment increased SUMOylated HIF-1α levels; the addition of purified SENP1C reversed this increase. Increased HIF-1α SUMOylation was also observed in an immunofluorescence assay (Supplementary Figure S2). Similarly, Mc treatment increased the SUMOylation of NAC1 in PC3 cells (Figure 3E, Supplementary Figure S3). Together, these data suggest that Mc treatment leads to the accumulation of SUMOylated proteins in PC3 cells.

### Mc inhibited the proliferation of PCa cells

**Figure 4:**
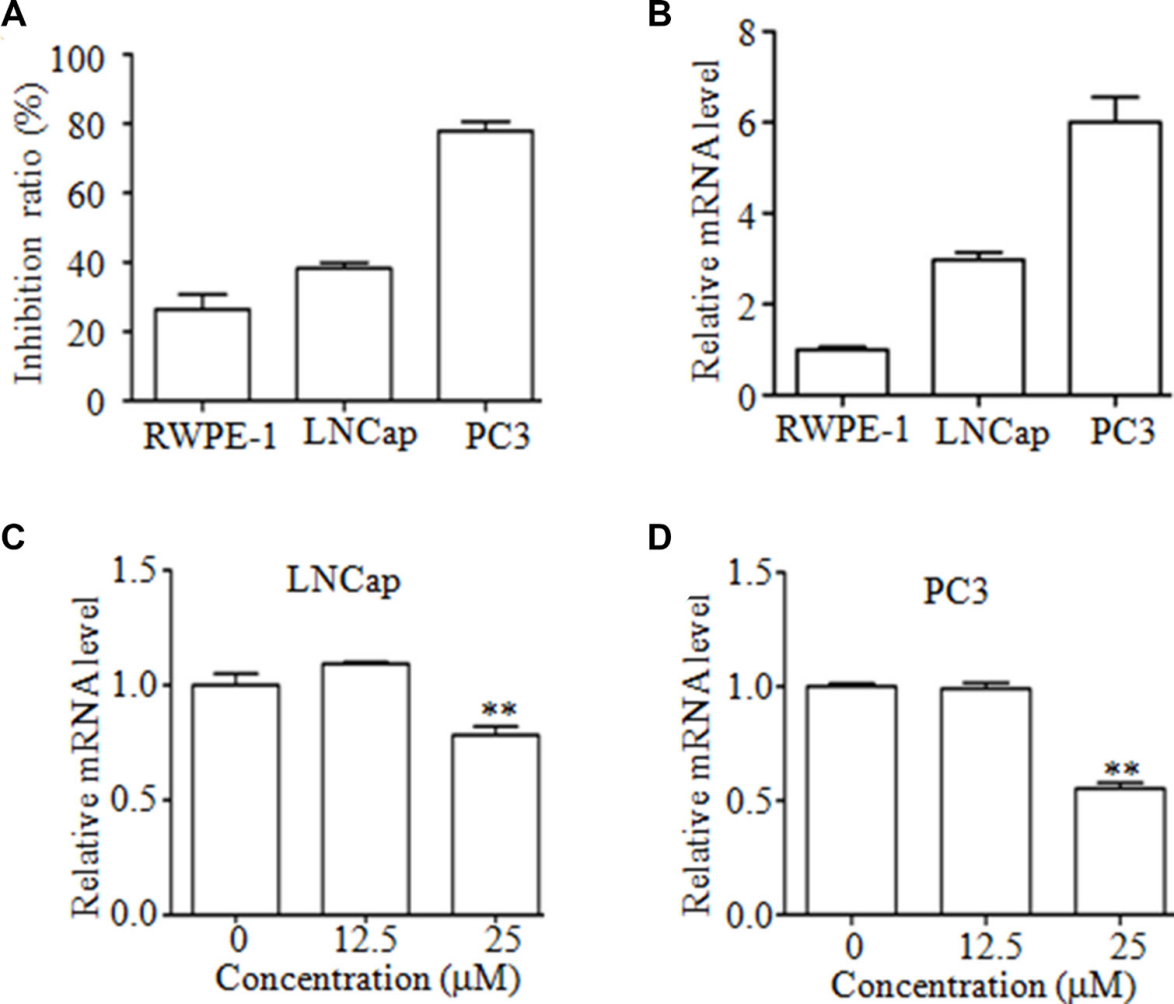
Mc inhibits the proliferation of PCa cells (**A**) PC3, LNCaP, and RWPE-1 cells were treated with the indicated concentrations of Mc for 24 h, and cell proliferation was evaluated by CCK8 assay. (**B**) Expression of SENP1 mRNA in PC3, LNCaP, and RWPE-1 cells was examined by qRT-PCR. (**C**–**D**) LNCaP (C) and PC3 (D) cells were treated with Mc for 24 h and SENP1 mRNA expression was measured using qRT-PCR. **p < 0.01. All values represent means and bars represent S.D. of three independent experiments. All experiments were repeated three times.

Next, we determined the effects of Mc on proliferation in PC3 cells, LNCaP cancer cells, and normal prostate epithelial RWPE-1 cells. As shown in Figure 4A, these cells showed different sensitivities to Mc treatment. After treatment with 25 μM Mc for 24 h, the inhibition ratios for PC3, LNCaP, and RWPE-1 cells were 78.00% ± 0.03, 38.33% ± 0.02, and 26.49% ± 0.04, respectively. Interestingly, higher SENP1 mRNA levels were associated with higher sensitivity to Mc in these cells (Figure 4B). These results indicate that prostate cancer cells may be more sensitive to Mc treatment than their normal counterparts. Moreover, Mc slightly, but significantly, decreased SENP1 mRNA levels in LNCaP and PC3 cells (Figure 4C and 4D).

### SENP1 contributes to Mc-induced inhibition of proliferation in prostate cancer cells *in vitro*

**Figure 5:**
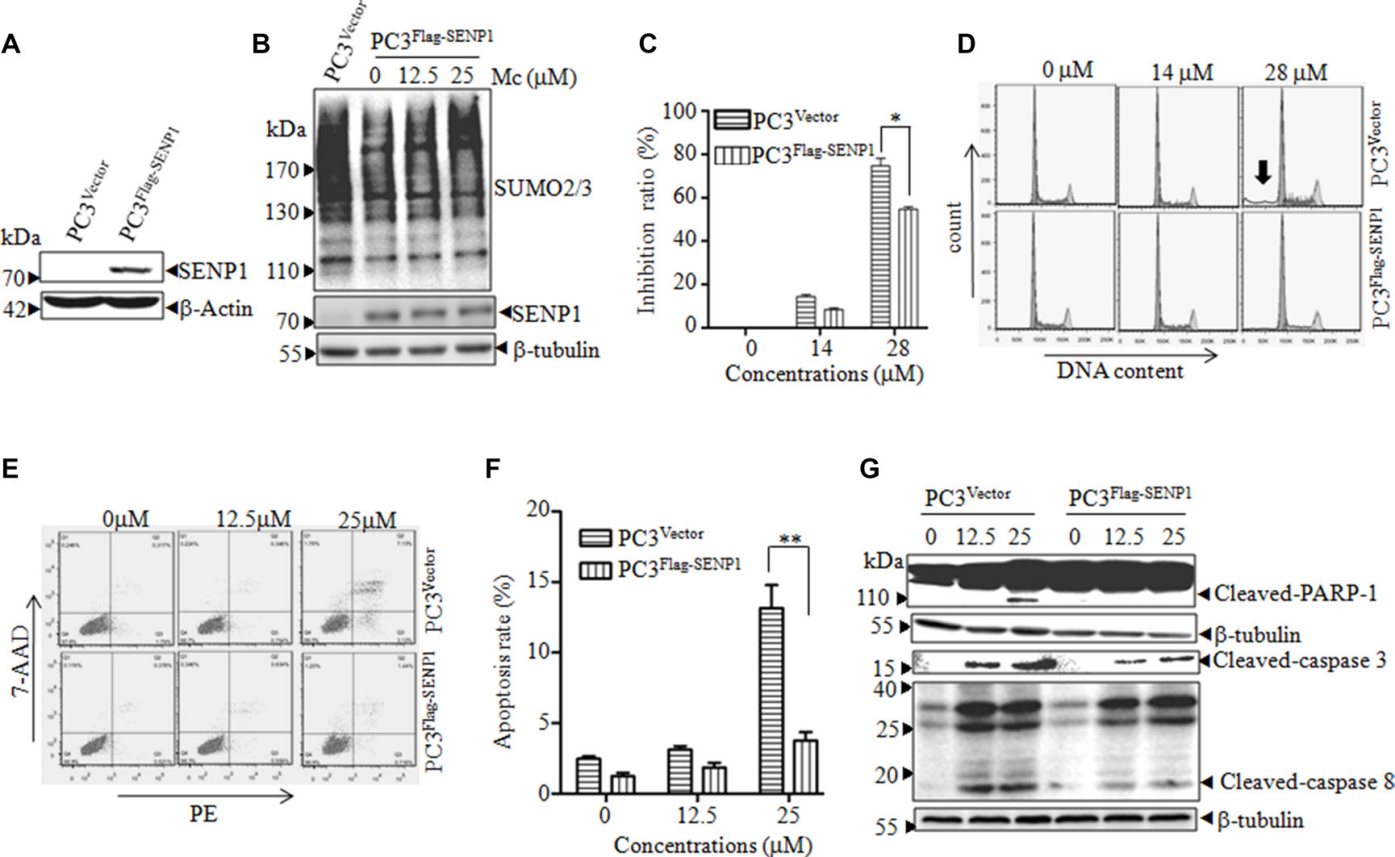
SENP1 overexpression inhibits Mc-induced apoptosis in PC3 cells (**A**) PC3 cells were stably transfected with empty vector (PC3^Vector^) or pBabe-Flag-SENP1 (PC3^Flag-SENP1^). The indicated proteins were detected by western blot. (**B**) PC3^Flag-SENP1^ cells were treated with Mc for 2 h, and SUMOylated protein was detected by western blot. (**C**–**G**) PC3^Vector^ and PC3^Flag-SENP1^ cells were treated with the indicated doses of Mc for 24 h. Cell proliferation was monitored with the CCK8 assay (C). Cell cycle distribution (D, arrow indicates Sub-G1 peak) and apoptosis (E, F) were analyzed by flow cytometry. The indicated proteins were examined by western blotting (G). All values represent means and bars represent S.D. of three independent experiments. All experiments were repeated three times. *p < 0.05, **p < 0.01.

SENP1 inhibition reduces the proliferation of prostate cancer cells [32]. Since Mc interacts with SENP1 and inhibits proliferation in prostate cancer cells, SENP1 inhibition may contribute to the Mc-induced reduction in proliferation. To investigate this possibility, PC3 cells were stably transfected with pBabe-Flag-SENP1 (PC3^Flag-SENP1^) or vector (PC3^Vector^) (Figure 5A). Overexpression of SENP1 decreased SUMOylated protein levels, and this effect was partially reversed by Mc treatment (Figure 5B). Moreover, overexpression of SENP1 reversed Mc-induced proliferation inhibition (Figure 5C). The inhibition rates for PC3^Vector^ and PC3^Flag-SENP1^ were 74.57% ± 0.04 and 54.53% ± 0.01, respectively (*p* < 0.05).

To investigate the mechanism by which Mc inhibited proliferation, we performed cell cycle analysis in PC3^Flag-SENP1^ and PC3^Vector^ cells treated with or without Mc. As shown in Figure 5D, Mc treatment (28 μM) increased the Sub-G1 and G2/M populations in PC3^Vector^ cells. Overexpression of SENP1 inhibited the Mc-induced increase in the Sub-G1 cell population (Table 1 and Figure 5D). We then analyzed the influence of Mc on cell apoptosis using a PE-Annexin-V/7AAD staining assay. Mc treatment increased the number of apoptotic PC3^vector^ cells, and overexpression of SENP1 inhibited Mc-induced apoptosis (Figure 5E and 5F). Consistent with these results, Mc-induced activation of caspase-3, caspase-8, and PARP cleavage were also reversed by overexpression of SENP1 (Figure 5G). These data suggest that SENP1 inhibition contributes to Mc-induced apoptosis in PC3 cells.

**Table 1:** SENP1 overexpression inhibits Mc-induced increases in Sub-G1 PC3 cell populations

(A) Cell cycle distribution of PC3^Vector^ cells treated with Mc for 24 h				
Mc (μM)	Sub-G1 phase (%)	G1 phase (%)	S phase (%)	G2 phase (%)
0	1.69 ±2.43	54.64 ±1.98	29.86 ±2.01	13.81 ±0.37
14	1.23 ±2.52	56.85 ±2.06	27.37 ±1.64	14.55 ±1.82
28	18.86 ±2.12^**^	46.08 ±1.86	18.77 ±1.97	16.29 ±1.04

(B) Cell cycle distribution of PC3^Flag-SENP1^ cells treated with Mc for 24 h				
Mc (μM)	Sub-G1 phase (%)	G1 phase (%)	S phase (%)	G2 phase (%)
0	0.41 ±2.27	55.44 ±2.18	26.78 ±1.32	17.37 ±1.53
14	0.33 ±2.54	55.59 ±1.6	27.17 ±1.75	16.91 ±2.3
28	11.50 ±2.09^**^	53.55 ±1.55	17.81 ±1.49	17.14 ±2.4

PC3^Vector^ and PC3^Flag-SENP1^ cells were treated with Mc for 24 h and cell cycle distribution was analyzed using flow cytometry.

** *p* < 0.01 compared to control cells.

### Mc suppresses PC3 tumor xenograft growth *in vivo*

**Figure 6:**
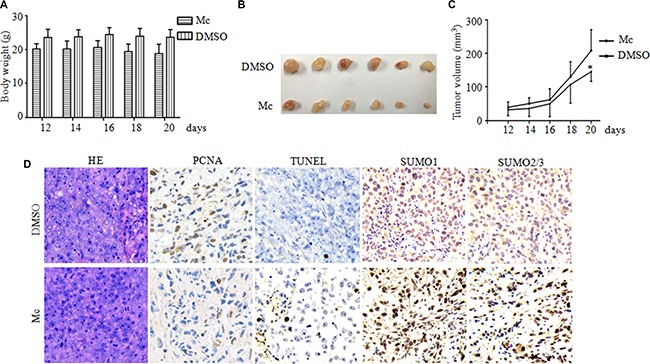
Mc suppresses PC3 tumor growth in a mouse xenograft model PC3 cells (5.6 × 10^6^ per mouse) were injected into five-week-old nude mice. When solid tumors grew to about 50 mm^3^, the mice received intraperitoneal injections of either vehicle control or Mc (10 mg/kg) daily for 20 days. Tumor sizes and body weights were measured every two days. (**A**) Effect of Mc on mouse body weight. (**B**) Images of xenograft tumors treated with Mc and controls on day 20. (**C**) Effect of Mc on xenograft tumor volume. *p < 0.05. (**D**) Expression patterns of PCNA, TUNEL, SUMO1 and SUMO2/3 were examined by immunohistochemistry in the xenograft tumors on day 20 in each group. Original magnification, w400.

To determine the efficacy of Mc *in vivo*, we conducted a xenograft study. PC3 cells were implanted subcutaneously in Balb/c nude mice. When the tumors became palpable, mice received intraperitoneal injections of either vehicle control or Mc at 10 mg/kg daily for 20 days. Body weights and tumor sizes were measured every two days. A slightly decrease in body weight was observed in the Mc-treated group compared to the control group (Figure 6A). On day 20, xenograft tumors treated with Mc (Figure 6B and 6C) were smaller than those in the control group (*p* < 0.05), indicating that Mc had a potent anti-tumor effect *in vivo*. This effect might be due to inhibition of cell proliferation and increased cell death, as revealed by decreases in PCNA staining and increases in TUNEL-positive cell numbers (Figure 6D). Consistent with an inhibition of SENP1 activity, Mc treatment also lead to the accumulation of SUMO1- and SUMO2/3-modified proteins in PC3 tumor xenografts (Figure 6D). These results suggest that Mc has anti-prostate cancer activity *in vivo*.

## DISCUSSION

SENP1 expression is elevated in prostate cancer specimens [32] and correlates with prostate cancer aggressiveness and recurrence [14]. Targeting SENP1 may be a novel approach for the treatment of prostate cancer. In this study, we demonstrate that Mc, a natural triterpenoid, is a novel SENP1 inhibitor with anti-prostate cancer activity *in vitro* and *in vivo*.

Mc is a saponin found in Chinese and Japanese natural medicines, such as the fruit of *Kochia scoparia* (L.), which has been used for centuries to treat pruritus, prostatitis, and irritability. Recently, it has been shown that Mc has anti-cancer activity [25]. However, the underlying mechanisms of these effects are largely unknown. In this study, we demonstrated that the anticancer activity of Mc may be due, at least in part, to its ability to inhibit SENP1. Indeed, Mc inhibited SENP1 activity in a gel-based assay *in vitro* and directly interacted with SENP1 in cells in the CETSA assay. Interestingly, Mc increased the thermal stability of full-length SENP1 in cells, but decreased the thermal stability of purified SENP1C *in vitro*. There are two possible explanations for this discrepancy. First, full-length SENP1 was used in the former assay while the core catalytic domain of SENP1 was used in the latter. Second, the proteins with which SENP1 interacts in cells may affect Mc-induced conformational changes in SENP1. Mc treatment also lead to the accumulation of SUMO-modified proteins in prostate cancer cells. This result was further confirmed by increases in SUMOylation of HIF-1α and NAC1, two known SUMO substrates, after Mc treatment. Finally, overexpression of SENP1 partially blocked Mc-induced inhibition of proliferation. Together, these results suggest that SENP1 is a novel target in the treatment of prostate cancer and demonstrate for the first time that Mc has anti-prostate cancer activity.

Triterpenoids are natural products containing 30 carbon atoms and are biosynthetically derived via the cyclization of squalene. Various biological, pharmacological, and therapeutic activities, including anti-inflammatory and anticancer effects, have been reported for an increasing number of triterpenoids [33–35]. Mc is a pentacyclic terpenoid, and this is the first report to show that pentacyclic terpenoids may be useful in the development of SENP1 inhibitors. Interestingly, Huang *et* *al*. reported that celastrol, another pentacyclic terpenoid, induced the accumulation of SUMOylated proteins in PC3 cells [36]. It is likely that celastrol also directly inhibits SENP1 activity. To date, approximately 20,000 triterpenoids have been identified in various parts of medicinal plants, and pentacyclic triterpenoids have the most potent anti-inflammatory and anti-cancer activities. Further investigation of Mc and similar compounds may lead to the development of novel, potent SENP1 inhibitors.

To our knowledge, this is first report demonstrating that Mc induces cell cycle arrest and apoptosis to inhibit prostate cancer growth. Mc treatment increased the Sub-G1 phase cell population and numbers of annexin-V positive cells, indicating an increase in apoptosis. Consistent with this result, Mc treatment increased levels of active caspase-3 and caspase-8 fragments and PARP1 cleavage. Mc also induced cell cycle arrest by reducing cyclin B and CDK1 levels. Inhibition of CDK1/cyclin B activity may block G2 to M phase transition, which in turn leads to arrest in the G2 phase. In support of this hypothesis, Mc treatment also suppressed phosphorylation of histone H3 on Ser10, indicating G2 arrest. Interestingly, overexpression of SENP1 largely blocked Mc-induced apoptosis, but only slightly decreased Mc-induced G2 arrest. Thus, SENP1 may play an important role in Mc-induced cell death in prostate cancer cells. However, the downstream effectors of SENP1 that mediate Mc-induced apoptosis are currently unknown and warrant further investigation.

In conclusion, we demonstrated that Mc is a novel SENP1 inhibitor that suppresses PC3 cell proliferation *in* *vitro* and *in vivo*. Mc may therefore serve as a promising template for developing novel, potent, and selective SENP1 inhibitors.

## MATERIALS AND METHODS

### Antibodies and reagents

Antibodies against vinculin (sc-7649) were purchased from Santa Cruz Biotechnology (Santa Cruz, CA). Anti-Flag M2 beads (M8823) and antibodies against Flag (F1804) and HA (H6908) were from Sigma-Aldrich. Antibody against RGS-His (34610) was purchased from Qiagen. SUMO1 (#4930), SUMO2/3 (#4971), PARP-1 (#9532), cleaved-caspase 3 (#9661), and cleaved-caspase 8 (#18C8) were purchased from Cell Signaling Technology. Anti-SENP1 (ab108981) was from Abcam. Lipofectamine 2000 (11668-019) was purchased from Invitrogen. Momordin Ιc (purity > 98%, CAS:96990-18-0) was purchased from Chengdu Finn Biotechnology Co., LTD.

### Cell culture

RWPE-1, a non-neoplastic human prostate epithelial cell line, was maintained in K-SMF (Life Technologies, USA) supplemented with 5 ng/mL epidermal growth factor (EGF) and 50 μg/mL bovine pituitary extract. HEK293T cells were cultured in Dulbecco's Modified Eagle's Medium (DMEM, Hyclone), and PC3 and LNCaP cells were grown in RPMI-1640 medium (Sigma-Aldrich) with 10% fetal bovine serum (FBS, Gibco BRL) in a 5% CO_2_/95% air humidified atmosphere at 37°C. PC3 cells stably transfected with pBabe-Flag-SUMO1/2/3, pBabe-Flag-SENP1, or vector were established and cultured as described previously [16].

### Western blotting

Cells were washed with PBS and lysed with lysis buffer (50 mM Tris-HCl, pH 6.8, 100 mM DTT, 2% SDS, 10% glycerol). Cell lysates were centrifuged at 20,000 g for 10 min, and proteins in the supernatants were quantified. Protein extracts were equally loaded onto 8–12% SDS−polyacrylamide gels, electrophoresed, and transferred to a nitrocellulose membrane (Bio-Rad). The blots were stained with 0.2% Ponceau S red to ensure equal protein loading. After blocking with 5% nonfat milk in PBS, the membranes were probed with antibodies. The signals were detected with a chemiluminescence phototope-HRP kit (Cell Signaling) according to the manufacturer's instructions. As necessary, blots were stripped and reprobed with anti-β-actin (Calbiochem) or β-tubulin (Sigma-Aldrich) antibody as an internal control. All experiments were repeated three times.

### *In vitro* gel-based SENP activity assay

SENP1C (20 nM) was incubated with compounds for 10 min at 37°C in reaction buffer (50 mM Tris-HCl pH 8.0, 20 mM NaCl, 2 mM CaCl_2_, 2 mM DTT). SUMO2-ΔRanGAP1 (1 mM final concentration) was then added followed by incubation for another 30 min at 37°C. The reaction was terminated by adding loading buffer and boiling on a heat block. The proteins were separated by 12% SDS-PAGE and visualized with coomassie brilliant blue (G250).

### Cellular thermal shift assay (CETSA)

1 × 10^7^ PC3 cells were collected and washed with ice-cold PBS three times. One mL of ice-cold PBS with Roche complete EDTA-free protease inhibitor cocktail (1:100) was then added to resuspend the cells, followed by three snap-freeze cycles consisting of 30 sec to 1 min in liquid nitrogen and then at 25°C in a thermal cycler or heating block until thawed. Cell lysates were then centrifuged at 20,000 g for 20 min at 4°C to pellet cellular debris.

To determine melting curves, cell lysates were divided into two aliquots; one was treated with Mc and the other with the corresponding concentration of DMSO (control). After 30 min of incubation at room temperature, the lysates were divided into smaller (35 μL) aliquots and heated individually at different temperatures (45, 47.1, 50.3, 54.4, 59.9, 64.2, or 67.1°C) for 3 min, followed by cooling for 3 min at room temperature. The heated lysates were centrifuged at 20,000 g for 20 min at 4°C in order to separate the soluble fractions from precipitates. The supernatants were transferred to new microtubes and analyzed by SDS-PAGE followed by western blots.

The procedure for establishing isothermal dose-response curves was similar to that for melting curves, except that the compound concentration rather than temperature was varied. All cells were heated at 60°C, which was determined based on analysis of the data obtained during melting curve experiments.

The above procedures were modified when purified SENP1C protein was used for the thermal shift assay. Briefly, for the thermal melt curve, 4 μg of purified SENP1C per reaction was incubated with 50 μM Mc or DMSO for 30 min at room temperature and then heated individually at different temperatures (30, 32, 35.2, 39.3, 44.9, 49, or 51.9°C) for 3 min. The temperature used for the isothermal dose-response fingerprint assay was 45°C. Otherwise, this procedure was conducted as described above.

### Immunoprecipitation

PC3 cells were transfected with plasmids encoding HA-SUMO2 and Flag- NAC1 or Flag-HIF-1α. Forty-eight hours later, cells were treated with 20 μM Mc for 2 hours (cells transfected with Flag-HIF-1α were pretreated with 5 μM MG132 for 4 hours) and lysed in 1 mL RIPA (50 mM Tris–HCl, pH 7.4, 150 mM NaCl, 1 mM EDTA, 1% Triton X-100, 2 mM NEM, cocktail (1:100)). Cell lysate was incubated with anti-Flag M2 beads at 4°C overnight, and beads were washed with RIPA (containing 2 mM NEM) 5 times. Immunoprecipitated proteins were divided equally into two parts. One was denatured with loading buffer, and the other was incubated with purified SENP1C as described for the *in vitro* gel-based SENP activity assay. Samples were analyzed by immunoblotting.

### Real-time quantitative PCR

Total RNA was isolated using the TRIzol kit (Invitrogen). Complementary DNA was synthesized using the cDNA synthesis kit (Transgen) according to the manufacturer's instructions. Fluorescence real-time RT-PCR was performed with FastStart Universal SYBR Green Master (ROX). Pairs of PCR primers used to amplify the target genes were as follows: SENP1, 5′-ATCAGGCAGTGAAACGTTGGAC-3′ (forward) and 5′-GCAGGCTTCATTGTTTATCCCA-3′ (reverse); GAPDH, 5′- CCACTCCTCCACCTTTGAC -3′ (forward) and 5′- ACCCTGTTGCTGTAGCCA -3′ (reverse). The experiment was performed in triplicate and repeated at least three times.

### Cell proliferation assay

Mc-induced inhibition of cell proliferation was measured using the Cell Counting Kit-8 assay kit (Dojindo, Kumamoto, Japan). Cells (1 × 10^4^) were seeded and grown overnight on a 96-well plate and then treated with different concentrations of Mc. After incubation for 24 h, 10 μL of CCK8 was added to each well. After incubation for another 2 h, the absorbance at 450 nm was measured using a Synergy H4 Hybrid Microplate Reader. The cell proliferation inhibition ratio was calculated with the following formula: cell proliferation inhibition ratio (%) = (OD Control–OD treated)/OD control × 100%.

### Cell cycle analysis

Cell cycle analysis was performed by measuring PI staining. In brief, PC3^Vector^ and PC3^Flag-SENP1^ were treated with Mc (0, 14, or 28 μM) for 24 h. Cells were then collected, washed with ice-cold PBS, and fixed in 75% ethanol for 24 h at −20°C. Cells were then washed twice with cold PBS, treated with RNAase (10 μg/mL) for 30 min at 37°C to remove RNA, followed by staining with PI (50 μg/mL) before analysis with flow cytometry. The percentages of cells in the Sub-G1, G0/G1, S, and G2/M phases were calculated. All experiments were repeated three times.

### Cell apoptosis analysis

Apoptosis was assessed by examining the binding of annexin V-PE to phosphotidylserine, which was externalized to the outer leaflet of the plasma membrane. PC3^Vector^ and PC3^Flag-SENP1^ cells (about 4 × 10^5^) were seeded on a six-well plates for 24 h. Cells treated with or without Mc (25 μM) for 24 h were resuspended in the binding buffer provided in the annexin V-PE/7-AAD apoptosis detection kit (559763, BD) and incubated with 5 μL of annexin V-PE reagent and 5 μL of 7-AAD for 15 min at room temperature in the dark. Stained cells were analyzed using flow cytometry. All experiments were repeated three times.

### Mouse xenograft assay

Five-week-old male nude mice (Balb/c) were purchased from Shanghai Slack Laboratory Animal Co., LTD, and maintained in a standard environment. The mice were allowed to acclimatize for at least 1 week prior to the experiment. PC3 cells (5.6 × 10^6^ per mouse) suspended in 0.2 mL of PBS were inoculated subcutaneously in the left flank of each mouse. When tumors became palpable, mice were divided randomly into control (*n* = 6) and treatment groups (*n* = 6). Mice in the treatment group received intraperitoneal injections of Mc at 10 mg/kg daily for 20 days while the control group received DMSO injections. Tumor sizes were measured using calipers, volumes were calculated using a standard formula (width^2^ ×length/2), and body weights were measured every two days. The xenograft tissues were collected immediately after the animals were sacrificed and stored at −80°C for further study.

### Immunohistochemical staining

Immunohistochemical studies were carried out on formalin-fixed, paraffin-embedded, 4 μm-thick tissue sections. The sections were deparaffinized and rehydrated, and antigen retrieval was performed using retrieval solution. Sections were then quenched with 0.3% hydrogen peroxide in methanol for 30 min to block endogenous peroxidase activity and washed in TBS (pH 7.2). Subsequently, sections were blocked with 5% normal goat serum for 20 min and then incubated with primary antibodies. For proliferation studies, the sections were stained with PCNA-specific antibody (Dako) [37]. Apoptotic cells were detected in sections using the TUNEL stain as described previously [38]. Global SUMOylation changes were detected with antibodies against SUMO1 and SUMO2/3. Subsequent counterstaining was performed with hematoxylin.

### Statistical analysis

The data are presented as the means ± SD of at least three independent experiments. Student's *t*-tests were performed using GraphPad Prism 5.0 software (GraphPad Software); *p* < 0.05 was considered statistically significant.

## SUPPLEMENTARY MATERIALS FIGURES


