# Supplementary material for: Momordin Ic, a new natural SENP1 inhibitor, inhibits prostate cancer cell proliferation
Source: Oncotarget. 2016 Jul 16;7(37):58995–9005. doi: 10.18632/oncotarget.10636 (PMC5312290; doi:10.18632/oncotarget.10636)
Supplement: Supplementary file 1 [file oncotarget-07-58995-s001.pdf]

Momordin Ic, a new natural SENP1 inhibitor, inhibits prostate cancer cell proliferation

Supplementary Materials

SUPPLEMENTARY MATERIALS AND METHODS

Reagents

Antibodies against CDK1 (sc-53219), cyclin B (sc-25764) and p-Histone H3 (Ser 10) (sc-8656-R) were purchased from Santa Cruz Biotechnology (Santa Cruz, CA). Antibodies against Flag (F1804) and HA (H6908) were purchased from Sigma-Aldrich.

Drug affinity responsive target stability (DARTS)

DARTS is a general methodology for identifying and studying protein-ligand interactions [1] and was performed to investigate the Mc-SENP1C interaction. Purified SENP1C protein was incubated with drugs or vehicle for 50 min at room temperature. The protein was then digested by adding pronase (10 mg/ml stock solution in water - aliquots stored at −20°C) dissolved in TNC buffer (50 mM Tris-HCl pH 8.0, 50 mM NaCl, and 10 mM $CaCl_2$) to the mixture at an appropriate ratio and incubating for 30 min. The reaction was stopped by adding concentrated SDS-PAGE loading buffer to a final 1 × concentration, mixing well and immediately boiling. The samples were then subjected to SDS-PAGE and stained with Coomassie brilliant blue.

Immunofluorescence

PC3 cells grown on glass coverslips were transfected for 48 h with plasmids encoding HA-SUMO2 and Flag-NAC1 or HIF-1α. The cells were then treated with 20 μM Mc for 2 h (cells transfected with Flag-HIF-1α were pretreated with 5 μM MG132 for 4 hours) and fixed with 4% paraformaldehyde for 30 min at room temperature. After washing the cells three times with phosphate-buffered saline (PBS), they were incubated in 0.2% Triton X-100 for 15 min at 4 °C and washed again with PBS for three times. The cover slides were then carefully dried, encircled using a Gene-Tech Pen and blocked with 2% BSA in PBS for 1 h at room temperature on a shaker. For co-localization assays, cells were incubated first with a mixture of anti-Flag and anti-HA antibodies overnight at 4°C and then with secondary antibody (FITC-labeled secondary antibody for Flag, TRITC-labeled secondary antibody for HA) for 1 h at room temperature. DAPI was added on the cover slides before they were mounted on slides. Images were captured using Nikon confocal laser scanning microscope.

REFERENCES

1. Lomenick B, Jung G, Wohlschlegel JA, Huang J. Target identification using drug affinity responsive target stability (DARTS). Curr Protoc Chem Biol. 2011; 3:163–180.

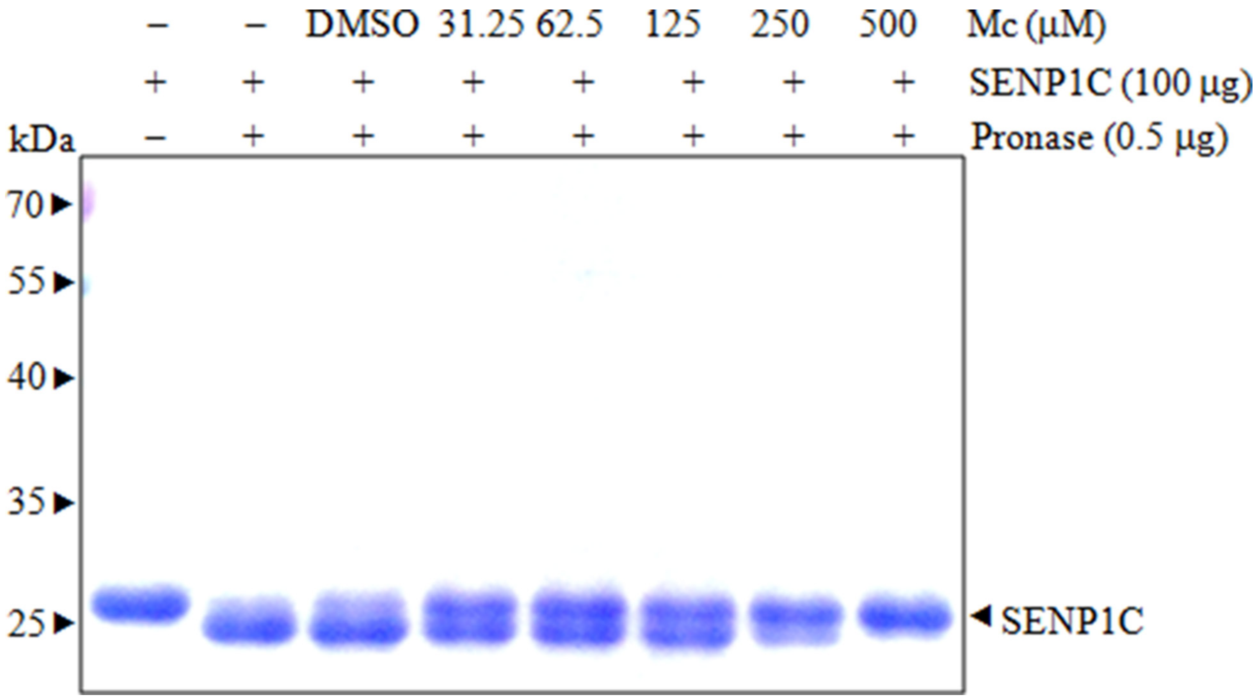

Supplementary Figure S1: Mc interacts with SENP1C *in vitro*. Purified SENP1C proteins were incubated with Mc followed by digestion with pronase, as described for DARTS assays in the "Supplementary Materials and Methods". The degree of SENP1C degradation was evaluated based on Coomassie brilliant blue staining.

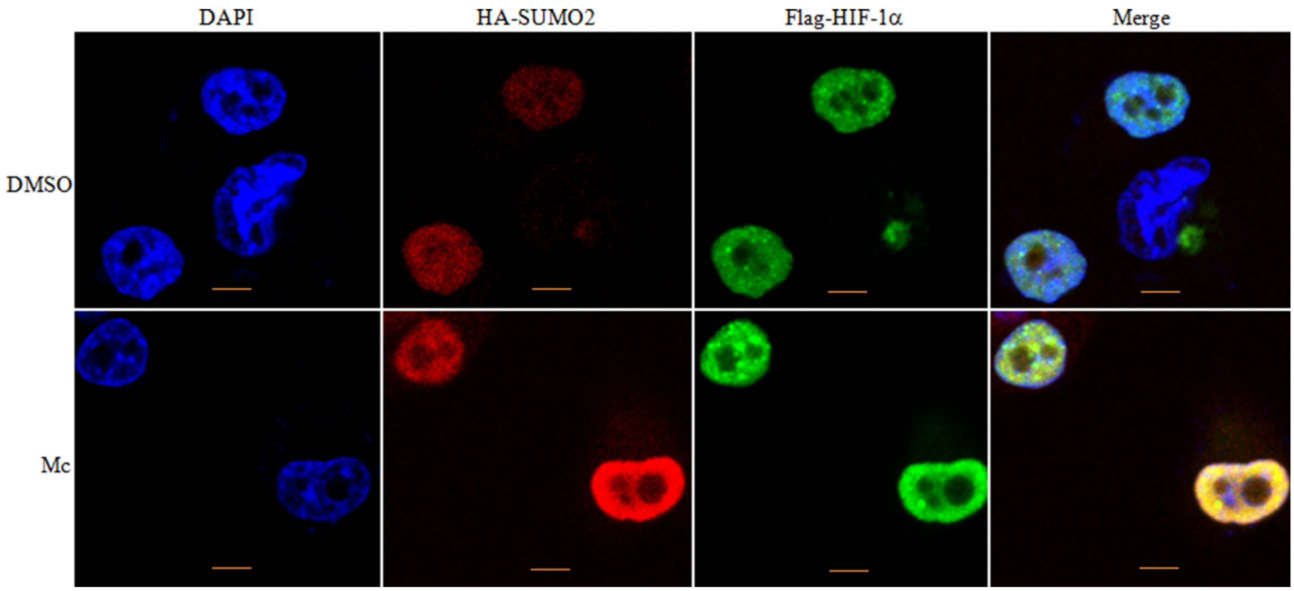

Supplementary Figure S2: Mc increases SUMOylation of HIF-1α in PC3 cells. PC3 cells grown on glass coverslips were transfected for 48 h with plasmids encoding HA-SUMO2 and Flag- HIF-1α. The cells were then pretreated with MG132 for 4 h followed by 20 μM Mc for 2 h. Colocalization of Flag-HIF-1a and HA-SUMO2 was detected in an immunofluorescence assay as described in "Supplementary Materials and Methods".

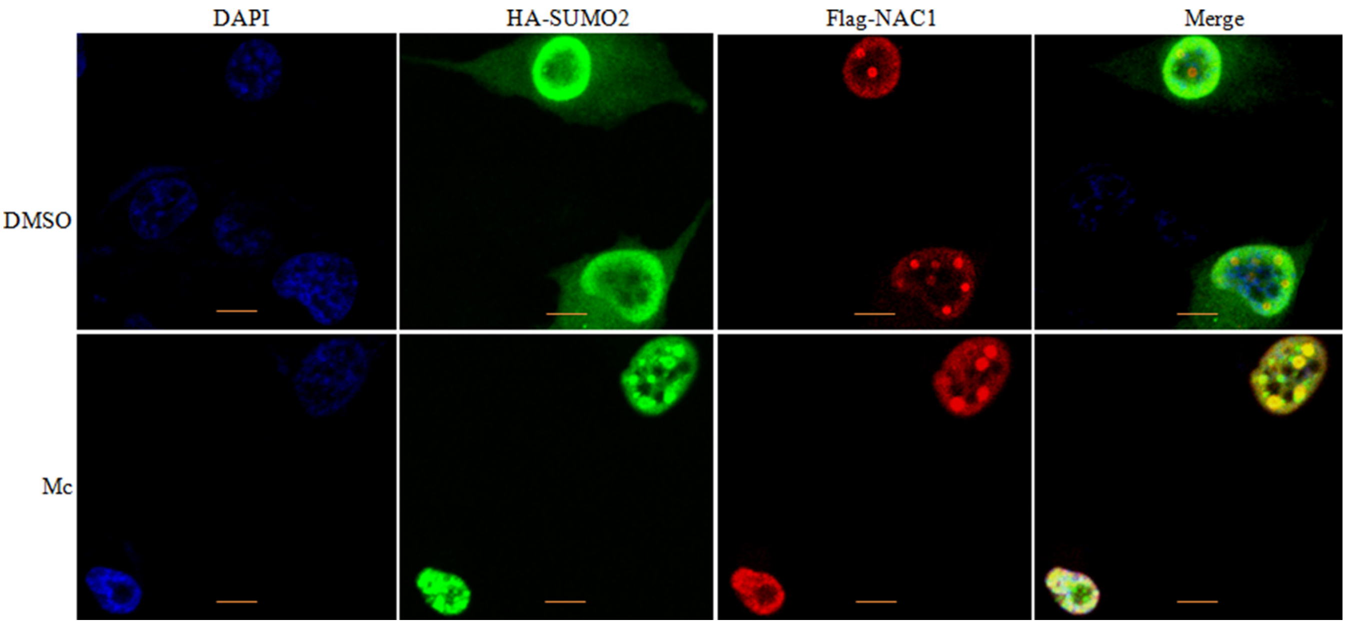

Supplementary Figure S3: Mc increases SUMOylation of NAC1 in PC3 cells. PC3 cells grown on glass coverslips were transfected for 48 h with plasmids encoding HA-SUMO2 and Flag-NAC1. The cells were then treated with 20 µM Mc for 2 h. Colocalization of Flag-NAC1 and HA-SUMO2 were detected in an immunofluorescence assay as described in “Supplementary Materials and Methods”.

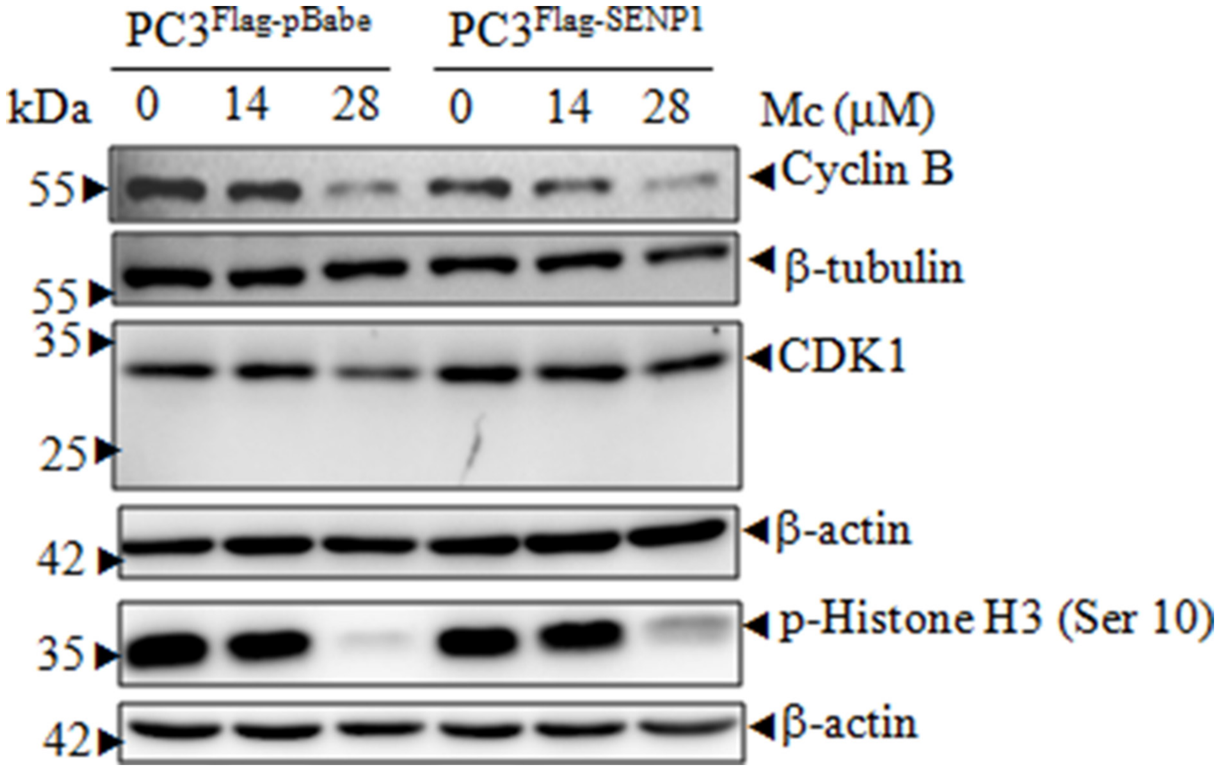

Supplementary Figure S4: Effect of Mc on the expression of cell cycle related proteins. $PC3^{Vector}$ and $PC3^{Flag\text{-}SENP1}$ cells were treated for 24 h with various concentrations of Mc, after which the indicated cell cycle-related proteins were detected by western blotting.
